# Day-Level Associations Between Substance Use and HIV Risk Behavior Among a Diverse Sample of Transgender Women

**DOI:** 10.1089/trgh.2018.0032

**Published:** 2018-12-26

**Authors:** Brett M. Millar, Devin English, Raymond L. Moody, H. Jonathon Rendina, Demetria Cain, Nadav Antebi-Gruszka, Joseph A. Carter, Jeffrey T. Parsons

**Affiliations:** ^1^ Center for HIV/AIDS Educational Studies & Training, Hunter College of the City University of New York (CUNY), New York, New York.; ^2^ Health Psychology and Clinical Science Doctoral Program, The Graduate Center of the City University of New York (CUNY), New York, New York.; ^3^ Department of Psychology, Hunter College of the City University of New York (CUNY), New York, New York.; ^4^ Community Health Sciences, University of Illinois-Chicago School of Public Health, Chicago, Illinois.; ^5^ Mental Health Counseling, Department of Psychology, City College of the City University of New York (CUNY), New York, New York.

**Keywords:** alcohol, drug use, harm reduction, intensive longitudinal data, prevention

## Abstract

**Purpose:** Transgender women in the United States face elevated rates of HIV and of substance use. Studies measuring overall or aggregate levels of substance use have linked use to increased HIV transmission risk behavior (TRB). Although intensive longitudinal studies in other populations have found day-level links between substance use and TRB, no study has yet explored such links among transgender women. This study aimed to fill this gap in the literature.

**Methods:** Utilizing survey and 60-day timeline follow-back interview data from a sample of 214 transgender women in New York City, we tested whether day-level heavy drinking, marijuana use, and/or nonprescription stimulant use were associated with odds of engaging in any sex (vs. no sexual activity) or engaging in TRB (vs. sex without TRB), adjusting for overall levels of use.

**Results:** Multilevel models showed that each of the three substance types was associated with greater odds of engaging in sex on a given day—and more strongly so for heavy drinking among those with higher rates of heavy drinking, and for stimulant use among those with lower rates of stimulant use. Only marijuana use was associated with greater odds of TRB on a given day, but only among those with higher rates of use.

**Conclusion:** These findings substantiate day-level links between substance use and engaging in sexual activity among transgender women, and importantly, between marijuana use and greater likelihood of TRB on a day when sexual activity occurs. This highlights the importance of addressing substance use for sexual health among transgender women especially focusing on marijuana use.

## Introduction

In efforts to better understand and address health inequities experienced by transgender women—defined as individuals assigned a male sex at birth who currently identify their gender as female—greater knowledge is needed regarding risk factors for adverse health outcomes.^1^ One prominent health inequity facing transgender women in the United States is elevated rates of HIV, with an estimated prevalence rate of 21.6%,^2,3^ likely rooted in discrimination and oppression, and involving high rates of poverty, unemployment, and lack of social support.^4–9^ Accordingly, the need for a greater understanding of risk factors influencing HIV transmission risk behavior (TRB) among transgender women is paramount.

Numerous cross-sectional studies have observed associations between overall or summed substance use indicators (e.g., averaged use over a given period, or any use vs. none) and rates of TRB among transgender women.^10–16^ This link between substance use and TRB is especially concerning given the findings of other studies showing that transgender women also have comparatively high rates of substance use.^17–21^

These cross-sectional findings resonate with extensive work that has focused on substance use as a risk factor in TRB among other populations such as heterosexual cisgender women and men^22,23^ and gay and bisexual men.^24,25^ However, research in these other populations has built upon these cross-sectional findings by studying associations between substance use on a given day and subsequent TRB events occurring on the same day.^26–28^ For example, Rendina et al.^27^ observed day-level links between heavy drinking, marijuana, and club drug use and sexual behavior among gay and bisexual men, whereby use of each substance (especially club drugs) was associated with (1) increased odds of engaging in any sexual activity (though not necessarily TRB), and (2) increased odds of engagement in TRB (vs. sex that did not involve TRB) on that day. By including the individual's overall or aggregated level of use, analyses were able to show that the day-level associations for club drug use and for marijuana were stronger among less frequent users compared with those who use more frequently. This kind of nuanced information highlights the value of intensive longitudinal analyses and is not possible in cross-sectional studies analyzing overall use. Unfortunately, day-level studies focusing on transgender women are scarce. To our knowledge, the only event-level study on substance use and TRB among transgender women is the study by Delgado et al. ^29^ in Peru that combined gay and bisexual men with transgender women and only asked about alcohol use and the most recent sex event with up to three partners in the past 90 days.

Accordingly, we aimed to address this gap in the literature by examining whether substance use on a given day was associated with engagement in any sex and/or with engagement in sex involving TRB, among a diverse sample of transgender women in New York City. Specifically, we aimed to test day-level associations between heavy drinking (defined as having five or more drinks in the one sitting), marijuana, or nonprescription stimulant drugs on sexual engagement and sexual TRB. These variables are given in Table 1. Given the comparatively high rates of substance use generally observed among transgender women, we aimed to adjust for the individual's average level of use for each of the three substance types and to test whether the individual's average level of use moderated the day-level associations for each outcome. We hypothesized that, on days of substance use, the positive associations between use and both sexual engagement and sexual TRB would be even greater among transgender women with comparatively lower overall rates of use, and that the associations would still remain significant, although less strong, among transgender women with comparatively higher overall rates of use.

**Table 1.** Summary of Study Predictors and Outcomes

**Day-level predictor variables** Heavy drinking on a given day Using marijuana on a given day Using stimulant drugs on a given day	**Day-level outcomes** The odds of engaging in any sex (vs. no sex) The odds of engaging in sexual TRB (vs. sex that does not involve TRB)
**Individual-level predictor variables** Overall frequency of heavy drinking Overall frequency of marijuana use Overall frequency of stimulant drug use	

TRB, transmission risk behavior.

## Methods

### Participants

Participants were 214 transgender women from the New York City metropolitan area who completed baseline visits for a substance use and sexual risk behavioral intervention tailored to transgender women, *Project T-Talk*, between May 2014 and September 2016. For recruitment, we adapted a mix of active, passive, and online strategies that we have previously used to recruit gay and bisexual men.^30–32^ Active strategies included collaborating with community-based organizations, visiting venues, bars, and nightclubs, and building interpersonal connections and trust within local transgender communities through meetings and events. Additional passive recruitment efforts included distributing study recruitment materials to health clinics specializing in medical, mental health, and substance use treatment for transgender women. Online recruitment efforts consisted of emailing LISTSERVS, sending Project Newsletter emails to transgender women who had expressed interest in participating and advertising on social networking websites (e.g., Facebook, Craigslist). We also provided drop-in hours to maximize flexibility for participants to attend the initial baseline visit throughout the day and evenings.

We screened a total of 487 transgender women, 382 (78%) of whom met the criteria for study eligibility. These criteria included being 18 years old or older, being able to complete a survey in English, being a transgender woman (i.e., assigned a male sex at birth and identifying as female at the time of participation), providing contact information, living in New York City metropolitan area, and reporting at least one sexual act or one day of drug use in the past 60 days. We deemed participants ineligible if they were currently enrolled in a substance abuse treatment or an HIV risk or substance use intervention. Of the 219 transgender women who attended the baseline assessment, 214 completed all measures of interest and were included in the analytic sample for this study. The baseline assessment lasted between 70 and 150 min and consisted of a survey that assessed demographic characteristics and psychosocial factors (e.g., depressive symptoms), and a timeline follow-back (TLFB) interview (Sobell and Sobell, 1992) that assessed sexual behavior and substance use in the past 60 days (Irwin et al., 2006). Participants received $40 for their participation in the baseline assessment. The Hunter College Institutional Review Board approved all study protocols.

### Measures

#### Demographics

Participants self-reported their race and ethnicity, gender identity (i.e., whether participants consider themselves to be a transgender woman or a woman of transgender experience), relationship status (i.e., whether they are currently seeing someone they consider to be a main partner), sexual orientation identity (coded as heterosexual vs. lesbian, gay, bisexual, or queer; LGBQ), HIV status (with an HIV-negative status confirmed by testing or an HIV-positive status confirmed with documentation), annual income (in brackets of $10,000), level of education, current age, and the age when the participant first began living as a woman.

#### Behavioral variables

Participants completed a 60-day TLFB interview,^33,34^ indicating substance use and sex that occurred over the 60 days before the baseline assessment. For the present analyses, we focused on daily reports of heavy drinking (i.e., having five or more alcoholic drinks), use of marijuana, and use of nonprescription stimulants (i.e., cocaine/crack, ecstasy, methamphetamine), in line with the analytic plan of Rendina et al.^27^ For each day, we created three dichotomous indicators of whether the participant had engaged in heavy drinking, marijuana use, or nonprescription stimulants, and used these variables as day-level (i.e., Level 1) indicators of substance use within our models. We also aggregated these three daily substance use indicators to the individual level to serve as global count variables indicating the number of days of use for each substance (i.e., Level 2).

For days on which a participant reported sex, we coded whether it was penetrative sex (i.e., involving vaginal or anal intercourse), whether condoms were used, whether it was with a casual partner or with a main partner who was of known discordant or unknown HIV status, and whether the event involved transactional sex (i.e., any exchange of sex for housing or money). We coded any act in which a participant had condomless penetrative sex with any casual partner and/or with a main partner of discordant or unknown HIV status as TRB. We did not collect information from all participants on whether they were using preexposure prophylaxis (PrEP) and also did not measure whether their partners were using PrEP, and were thus unable to include PrEP usage in our definition of TRB—however, PrEP use was very rare in the early stages of data collection and it is unlikely to have a substantial impact on results. To match the day-level substance use variables, we aggregated event-level sexual behavior data to the day level. Specifically, we created two variables to use as outcomes within our models: (1) a dichotomous indicator of whether any sexual activity occurred that day and (2) a trichotomous indicator of whether the participant engaged in no sex (coded as 0), sexual activity without TRB (e.g., mutual masturbation, oral sex, penetrative sex with a condom, condomless penetrative sex with a known seroconcordant partner; coded as 1), or sex involving TRB (coded as 2).

### Data analysis plan

We began by examining descriptive statistics to characterize the demographic makeup of the sample. Next, we examined Spearman nonparametric correlations between the aggregated substance use count variables (i.e., days of heavy drinking, days of marijuana use, and days of nonprescription stimulant use) and the aggregate number of days having sex and sex involving TRB. We then ran a series of multilevel models, with day-level dichotomous indicators of heavy drinking, marijuana use, and nonprescription stimulant use at Level 1 and individual-level aggregated frequencies of use for each substance as a count variable at Level 2. For the first outcome, whether or not participants engaged in any sexual activity on a given day, we used a binary logistic outcome (Model 1). For the second outcome, the trichotomous variable indicating whether the participant had no sex, had sex without TRB, or sex with TRB on a given day, we used a multinomial logistic model (Model 2). Although we used all data (both sex days and nonsex days) to calculate coefficients for Model 2, we focused our analysis on the portion of the model comparing sex days without TRB and TRB days. As such, we do not report comparisons with nonsex days in Model 2.

We ran both models with cross-level interactions between corresponding day-level and grand mean centered individual-level substance use (e.g., day-level heavy drinking by individual-level heavy drinking) to examine whether associations between day-level substance use and marginal probabilities for sexual engagement and TRB on a given day varied based on an individual's overall level of substance use. As such, these models included the following independent variables: day-level use of each of the three substance types (Level 1), aggregate individual-level use for each substance type (Level 2), and an interaction between corresponding substance categories on each level. We ran models with a random intercept, using an AR(1) covariance structure, and adjusted for HIV-positive status, relationship status, and day within the TLFB frame (i.e., 1 through 60 days). We also ran additional analyses adjusting for whether the sex event involved transactional sex to check if patterns remained consistent.

## Results

Demographic characteristics are given in Table 2. Approximately a third of the sample identified as Black/African American, 28% identified as Latina, a quarter identified as white, 13% identified as multiracial, and the remaining five participants identified as Asian. A majority of participants had an HIV-negative status (65.4%), had a yearly income below $20,000 (80.4%), and had an education level of some college or more (57.9%). Approximately half of the participants reported being partnered (49.1%) and LGBQ identified (52.8%). Marijuana was the most frequently used substance with a mean use of 20.5 days and a median use of 7.0 days. Heavy drinking days (*M*=5.4, *SD*=9.2, median=1) and nonprescription stimulant use days (*M*=4.5, *SD*=10.1, median=0) were relatively less frequent during the 60-day period for most participants. The mean number of sex days was 12.6 (*SD*=13.9) during the 60-day period, with 85.5% of participants reporting at least two or more sex days. The mean number of TRB days was 4.3 (*SD*=10.1), with 40.2% of participants reporting at least two or more TRB days.

**Table 2.** Demographic Characteristics and Prevalence of Outcomes Among Transgender Women in New York City (*N*=214)

	*n*	%
Race/ethnicity		
Black/African American	69	32.2
Latina/Latinx	60	28.0
White	52	24.3
Multiracial	28	13.1
Asian	5	2.3
Gender identity		
Transgender woman	208	97.2
Other	6	2.8
Relationship status		
Single	109	50.9
Partnered	105	49.1
Sexual orientation		
LGBQ	113	52.8
Heterosexual/straight	101	47.2
HIV status		
Negative/unknown	140	65.4
Positive	74	34.6
Income		
Below $20K	172	80.4
$20K or more	42	19.6
Education		
High school or less	90	42.1
Some college	62	29.0
Bachelor degree	49	22.9
Graduate degree	13	6.1
	*M*	*SD*
Age (years; range 18–65)	34.3	11.7
Age of first living as a woman (*n*=208)	22.4	10.0
Number of heavy drinking days (median=1.0)	5.4	9.2
Number of marijuana use days (median=7.0)	20.5	24.2
Number of stimulant use days (median=0.0)	4.5	10.1
Number of days of any sexual activity (median=8.0)	12.6	13.9
Number of sexual TRB days (median=1.0)	4.3	10.1

LGBQ, lesbian, gay, bisexual, queer.

### Aggregate-only analyses

Table 3 provides bivariate correlations between aggregated sex days, TRB days, substance use days, and two covariates: HIV status and relationship status. We found that the number of sex days was positively correlated with total heavy drinking days, total stimulant use days, and being partnered—but not with marijuana use days. The number of TRB days was correlated only with total number of sex days. Aggregate levels of use among the three substances were positively correlated with each other such that, if a participant used one substance more frequently, they were more likely also to do so for the other substances. Overall, marijuana use and stimulant use were more frequently common among HIV-positive participants. Relationship status was not associated with use of any of the three substances.

**Table 3.** Bivariate Spearman Correlations Between Totals (of the Previous 60 Days) of Sex Days, Sexual TRB Days, Substance Use Days, and Covariates

	1	2	3	4	5	6	7
1. No. of sex days	1						
2. No. of sexual TRB days	0.37^***^	1					
3. No. of heavy drinking days	0.28^***^	0.11	1				
4. No. of marijuana days	0.10	0.02	0.16^*^	1			
5. No. of stimulant drug days	0.30^***^	0.08	0.31^***^	0.18^**^	1		
6. HIV Status (Ref=HIV-negative)	0.07	0.09	−0.12	0.19^**^	0.17^*^	1	
7. Relationship status (Ref=Single)	0.17^*^	−0.12	−0.06	0.04	0.07	0.07	1

^*^ *p*≤0.05.

^**^ *p*≤0.01.

^***^ *p*≤0.001.

### Simultaneous models of aggregate-level and day-level substance use

Table 4 provides the two multilevel models predicting odds of sexual engagement versus no engagement (Model 1) and odds of sex with TRB versus sex without TRB (Model 2). In Model 1, day-level heavy drinking (adjusted odds ratio [*AOR*]=2.30, *p*<0.001), marijuana use (*AOR*=3.53, *p*<0.001), and stimulant use (*AOR*=9.10, *p*<0.001) were all associated with significantly greater odds of sexual engagement (vs. no sex) on a given day. However, in Model 2, only day-level marijuana use (*AOR*=2.69, *p*<0.001) was significantly associated with greater odds of engaging in TRB (vs. sex without TRB). Although day-level heavy drinking trended in the positive direction, suggesting greater odds of TRB, it did not reach statistical significance.

**Table 4.** Multilevel Models Utilizing Day-Level and Individual-Level Substance Use to Predict Sexual Engagement and Sexual Transmission Risk Behavior

	*Model 1*^a^			*Model 2*^b^		
	*No Sex vs. Sex*			*Non-TRB vs. TRB*		
	*b*	*AOR*	*95% CI*	*b*	*AOR*	*95% CI*
Intercept	−2.63	0.07^***^	0.05–0.11	−1.85	0.16^***^	0.07–0.34
Day	−0.01	0.99^***^	0.99–1.00	0.00	1.00	0.99–1.00
HIV-status (Ref=HIV negative)	0.17	1.18	0.71–1.97	0.32	1.37	0.60–3.12
In relationship (Ref=Single)	0.68	1.97^**^	1.23–3.16	−0.84	0.43^*^	0.20–0.93
Level 1: day-level effects						
Day-level heavy drinking	0.83	2.30^***^	1.75–3.02	0.32	1.38	0.84–2.27
Day-level marijuana use	1.26	3.53^***^	2.88–4.32	0.99	2.69^***^	1.64–4.41
Day-level stimulant use	2.21	9.10^***^	6.39–12.97	−0.19	0.83	0.46–1.51
Level 2: individual-level effects						
Frequency of heavy drinking	−0.02	0.98	0.95–1.01	0.00	1.00	0.95–1.05
Frequency of marijuana use	−0.01	0.99	0.97–1.00	−0.04	0.96^*^	0.93–0.99
Frequency of stimulant use	0.05	1.05^**^	1.02–1.08	0.04	1.04	0.99–1.08
Day×individual effects						
Day-level×frequency of heavy drinking	0.07	1.07^***^	1.05–1.10	−0.01	0.99	0.95–1.04
Day-level×frequency of marijuana use	−0.01	0.99	0.98–1.00	0.04	1.04^*^	1.00–1.08
Day-level×frequency of stimulant use	−0.05	0.95^***^	0.93–0.97	−0.03	0.97	0.94–1.01

^*^ *p*≤0.05.

^**^ *p*≤0.01.

^***^ *p*≤0.001.

^a^ Binary logistic regression.

^b^ Multinomial logistic regression (only one of two comparisons are given). All models were adjusted for HIV status and relationship status and also day of data collection (i.e., day of timeline follow-back cycle).

AOR, adjusted odds ratio.

Regarding individual-level effects, aggregate number of stimulant use days (*AOR*=1.05, *p*<0.01) was associated with greater odds of engaging in sex on a given day (Model 1), indicating that those with more days of stimulant use in the 60-day period generally reported more days of engaging in sexual activity. In addition, aggregate marijuana use (*AOR*=0.96, *p*<0.05) was associated with lower odds of engaging in TRB on a given day (Model 2). This indicates that those with more days of marijuana use in the 60-day period generally reported fewer events of TRB when sexual activity did occur.

**FIG. 1.**
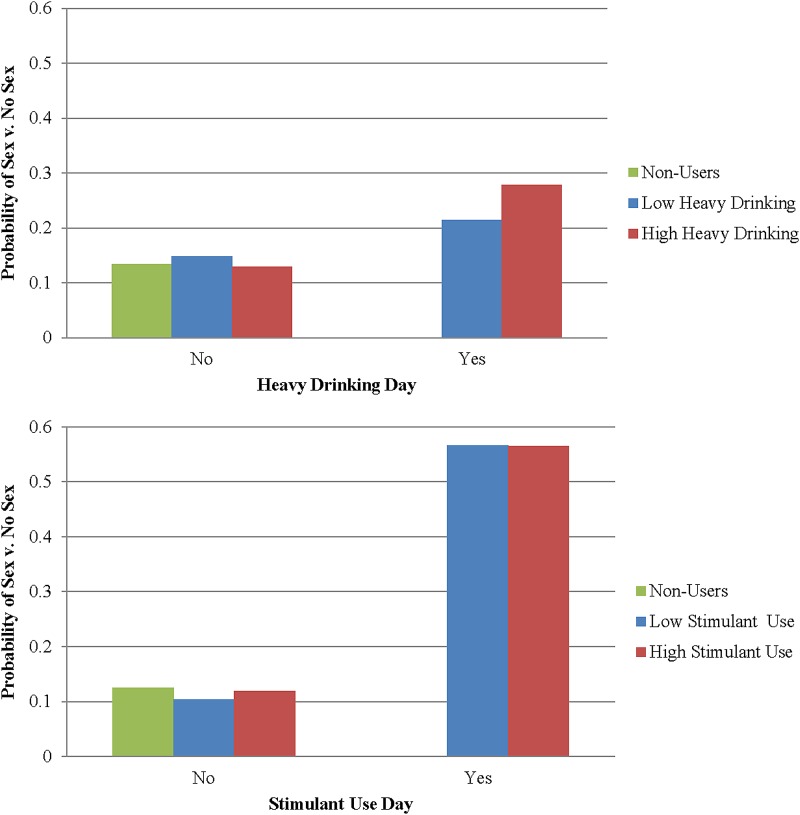
Marginal probabilities of engaging in sexual activity based on individual and day-level use of substances. Note: Low substance use and high substance use are defined at the 25th and 75th percentiles, respectively.

We next examined the interaction effects between day-level substance use and individual-level substance use. In Model 1, we found that individual-level heavy drinking moderated the association between day-level heavy drinking and odds of engaging in sex (*AOR*=1.07, *p*<0.001)—the positive association between day-level drinking and sexual engagement was higher among individuals who engaged in heavy drinking more often (Fig. 1). In the opposite direction, we found that individual-level stimulant use significantly moderated the association between day-level stimulant use and odds of engaging in sex (*AOR*=0.95, *p*<0.001), such that lower overall stimulant users had a greater increase in odds of sexual engagement on a day of use than those with more frequent use overall (Fig. 1).

**FIG. 2.**
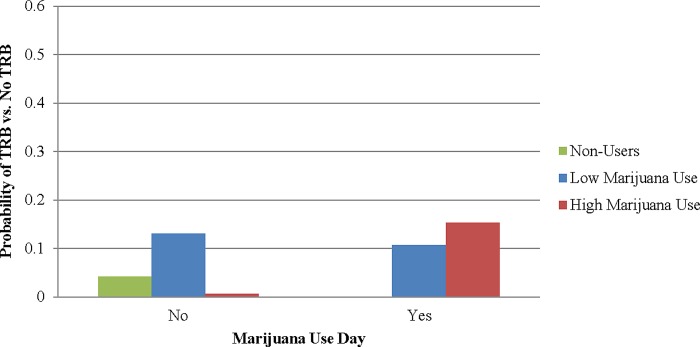
Marginal probabilities of engaging in sexual TRB based on individual-level and day-level use of substances. Note: Low substance use and high substance use are defined at the 25th and 75th percentiles, respectively. TRB, transmission risk behavior.

In Model 2, we found that individual-level marijuana use moderated the positive association between day-level marijuana use and odds of engaging in TRB (*AOR*=1.04, *p*<0.05). Although those with more frequent overall marijuana use tended to have lower odds of engaging in TRB on a sex day, the positive day-level association between marijuana use and their odds of TRB was stronger than for those with lower overall marijuana use (Fig. 2). Among those with lower overall marijuana use, the odds of engaging in TRB were not higher on a day of marijuana use versus a day of no marijuana use. The patterns were not meaningfully altered in additional analyses when we adjusted for whether the event involved transactional sex.

## Discussion

This study is, to our knowledge, the first to test for day-level associations between substance use (here, heavy drinking, marijuana use, and/or nonprescription stimulant use) and sexual behavior focusing on transgender women. We found that, as hypothesized, use of any of these three substance types on a given day was associated with greater odds of engaging in sexual activity on that day (compared with not engaging in sexual activity) among a diverse sample of New York City-based transgender women. Indeed, heavy drinking was associated with more than twice the odds of sexual engagement on a given day, marijuana use was associated with >3.5 times the odds of sexual engagement, and stimulant use was associated with >9 times the odds of sexual engagement on that day. These findings that substance use and sexual activity often co-occur for transgender women are consistent with research on samples of cisgender women^23^ and gay and bisexual men,^25,27^ suggesting that substance use on a given day was a more informative predictor of sexual engagement compared with overall levels of use.

Our hypotheses regarding day-level associations between substance use and greater odds of sexual TRB, however, were only partially supported. Only marijuana use on a given day was associated with greater odds of engaging in TRB compared with sex without TRB, at >2.5 times the odds. Although heavy drinking was in the hypothesized direction of increasing the odds of TRB, its local main effect did not reach statistical significance. The marijuana finding is consistent with evidence that marijuana use should be considered among the disinhibiting substances regarding sexual health,^35^ and suggests that interventions targeting the role of marijuana use on behaviors such as TRB among transgender women should be prioritized. However, the lack of significant findings for day-level heavy drinking and stimulant use predicting TRB in this study stands in contrast to previous studies—mostly examining overall frequencies of substance use—which have observed significant positive associations among transgender women^10,15,16^ and among other populations.^26–28^

Finally, we also tested whether these associations differed according to the individual's overall level of use. We found that the day-level association between heavy drinking and sexual engagement was stronger in those who engaged in heavy drinking more frequently, whereas the association between stimulant use and sexual engagement was stronger in those who engaged in less frequent use of stimulants overall. In terms of TRB, the day-level association between marijuana use and odds of TRB was stronger among those with greater overall marijuana use, counter to our hypothesis. These results suggest that the impact of marijuana on TRB on a given day is influenced by how frequently the substance is used by the individual in general.

This study begins to address a substantial gap in the literature on the influence of substance use on sexual behavior at the event-level among transgender women—and the findings have important implications in terms of HIV prevention efforts, particularly regarding marijuana use. Existing research on overall or aggregate substance use has found that transgender women who engage in more frequent use are at greater risk of engaging in TRB—suggesting that HIV prevention efforts should focus on transgender women who engage in more frequent substance use.^11–15^ Although our study cannot confirm the mechanism/s involved in the link between marijuana and TRB, our findings highlight the need to provide individuals frequently using marijuana with information and strategies to address links between their use and sexual risk behavior.

In terms of intervention implications, behavioral and motivational enhancement interventions have been shown to be effective at reducing substance use and sexual risk behavior among transgender women^36^ and gay and bisexual men.^37,38^ Some of these interventions incorporate a harm-reduction philosophy aimed at minimizing risk associated with substance use and sexual engagement. This study has implications for harm-reduction approaches to treatment for transgender women who may be resistant or ambivalent about totally eliminating their substance use. Among these, strategies may include reducing the quantity of use on a day of use, increasing self-efficacy for use of condoms or engaging in less risky sexual behavior, or increasing uptake of and adherence to medications (e.g., antiretroviral therapy; PrEP). The benefits of harm reduction on substance use and sexual health have been noted in other studies, such as a recent trial of a harm-reduction intervention that focused on gay and bisexual men that saw significant reductions in stimulant use, sexual partners, and receptive anal intercourse.^39^

The findings from this study should be considered in light of some limitations. First, the results of this study are based on a convenience sample who reported at least one recent sexual act or one recent drug use day, and thus these findings may not generalize to all transgender women. Second, this study utilized a TLFB interview to assess substance use and sexual behavior retrospectively. TLFB interviews are considered to be more accurate than other forms of retrospective recall but the risk of recall bias is still present.^40^ Although previous research has demonstrated relative consistency between retrospective and prospective assessments of substance use and sexual behavior,^27^ future research should examine these associations using prospective assessment methods (e.g., ecological momentary assessment). Third, day-level drinking did involve consideration of quantity of use, but our measures of day-level marijuana use and stimulant drug use did not ask participants about the quantity of their use on a given day. Fourth, our analyses were not able to adjust for PrEP use by the participants' partners or for the detectability of either person's viral load in each sexual event. Furthermore, our definition of sexual TRB mainly pertains to risk for HIV transmission and did not address the possibilities for sexually transmitted infections occurring through other types of sexual activity (classified in this study as non-TRB sexual engagement). Future research may seek to explore the role of substance use on these other sexual health outcomes.

## Conclusions

In sum, this study provides valuable, nuanced information about day-level links between substance use and sexual behavior among a sample of New York City-based transgender women. In part, our findings highlight the need to distinguish between different types of substances when addressing substance use in relation to sexual risk behavior among transgender women, given the differing patterns observed. In addition, the tailoring of harm-reduction and contingency-planning interventions according to the individual's overall level of use for each substance type is also recommended. Finally, it should be noted that a potential interpretation of our finding that both heavy drinking and stimulant use were positively associated with engaging in some sexual activity but not with engaging in TRB sex could be that, despite heavy drinking or stimulant use on a given day, many of the transgender women in our sample were able to navigate sexual activity with a partner without engaging in TRB. Further explorations of mechanisms and strategies used in these situations to prevent or avoid TRB may yield important insights for the sexual health of this population.

## Abbreviations Used

AOR: adjusted odds ratio
LGBQ: lesbian, gay, bisexual, or queer
PrEP: preexposure prophylaxis
TLFB: timeline follow-back
TRB: transmission risk behavior
